# Photoluminescence as a Complementary Tool for UV-VIS Spectroscopy to Highlight the Photodegradation of Drugs: A Case Study on Melatonin

**DOI:** 10.3390/molecules25173820

**Published:** 2020-08-22

**Authors:** Monica Daescu, N’ghaya Toulbe, Mihaela Baibarac, Alin Mogos, Adam Lőrinczi, C. Logofatu

**Affiliations:** 1 Lab. Optical Processes in Nanostructure Materials, National Institute of Materials Physics, Atomistilor str. 405 A, 77125 Bucharest, Romania; monica.daescu@infim.ro (M.D.); toulbe.nghaya@infim.ro (N.T.); lorinczi@infim.ro (A.L.); 2 Interdisciplinary School of Doctoral Studies, University of Bucharest, M. Kogalniceanu Blvd. no. 36-46, 050107 Bucharest, Romania; 3 S.C. Agilrom Scientific S.R.L., 77125 Bucharest, Romania; alin.mogos@agilrom.ro; 4 National Institute of Materials Physics, Atomistilor str. 405 A, 77125 Bucharest, Romania; constantinlogofatu@yahoo.com

**Keywords:** melatonin, photodegradation, photoluminescence

## Abstract

In this work, a complementary ultraviolet-visible (UV-VIS) spectroscopy and photoluminescence (PL) study on melatonin (MEL) hydrolysis in the presence of alkaline aqueous solutions and the photodegradation of MEL is reported. The UV-VIS spectrum of MEL is characterized by an absorption band with a peak at 278 nm. This peak shifts to 272 nm simultaneously with an increase in the band absorbance at 329 nm in the presence of an NaOH solution. The isosbestic point localized at 308 nm indicates the generation of some chemical compounds in addition to MEL and NaOH. The MEL PL spectrum is characterized by a band at 365 nm. There is a gradual decrease in the MEL PL intensity as the alkaline solution concentration added at the drug solution is increased. In the case of the MEL samples interacting with an alkaline solution, a new photoluminescence excitation (PLE) band at 335 nm appears when the exposure time to UV light reaches 310 min. A down-shift in the MEL PLE band, from 321 to 311 nm, as a consequence of the presence of excipients, is also shown. These changes are explained in reference to the MEL hydrolytic products.

## 1. Introduction

Melatonin (MEL) is generated in the mammalian pineal gland as a neurohormone [1]. It is a constituent of many pharmaceutical capsules [2] and is used to control the circadian rhythms and sleep cycle [3]. Until now, MEL has been shown to have strong anti-oxidant [4], anti-carcinogenic [5], and anti-inflammatory properties [6]. An important melatonin-induced effect is the blocking of the chain reaction of peroxidation by free radicals, such as OH•, NO•, ONOO^−^, and O_2_•^−^, which are generated during the anti-oxidation process [7]. The medical treatments in which MEL is most often recommended are those that require the control of blood pressure regulation and circadian rhythms and the reduction of anxiety before surgery, platelets in the blood, tardive dyskinesia by the diminution of the symptoms related to movement disorders, jet lag symptoms by the amelioration of coordinating alertness and movement, and insomnia [8,9,10,11,12,13]. As reported by Buscemi et al., MEL is rapidly adsorbed in the human body. After having a therapeutic effect related to sleep disorders, a part of the active compound is found in saliva, and another part is eliminated in urine [14]. In order to avoid the side effects induced in the presence of other drugs, such as a reduction of blood flow in the elderly [9], it was necessary to develop some monitoring techniques. Several techniques have been used for determining MEL in the absence and the presence of other drugs, such as high-performance liquid chromatography (HPLC) [13], cyclic voltammetry [15], spectrofluorimetric [16], and ultraviolet-visible (UV-VIS) spectrophotometry [17]. In the context of this progress, another topic of interest was the photodegradation of MEL in phosphate buffers containing 15% CH_3_OH with pH equal to 4, 7.4, and 9, which is a process monitored by HPLC [18]. The characterization of photoproducts was performed by mass spectroscopy, nuclear magnetic resonance (NMR), and Fourier-transform infrared (FTIR) spectroscopy. [18]. In comparison with these studies, our attention is focused on MEL hydrolysis in alkaline aqueous solutions and some photodegradation processes induced by ultraviolet (UV) light. For this purpose, new information about the chemical mechanisms of these reactions are reported using UV-VIS spectroscopy and photoluminescence (PL). The influence of excipients on MEL hydrolysis in the presence of alkaline aqueous solutions and the photodegradation process of this drug under UV light will also be shown. Arguments concerning the photodegradation products of MEL are shown by Raman scattering and FTIR spectroscopy. The solid-state reaction of MEL with NaOH, which was exposed to UV light, is also investigated by X-ray diffraction (XRD) and X-ray photoelectron spectroscopy (XPS).

## 2. Results and Discussion

### 2.1. Photodegradation of MEL Highlighted by UV-VIS Absorption Spectroscopy

**Figure 1:**
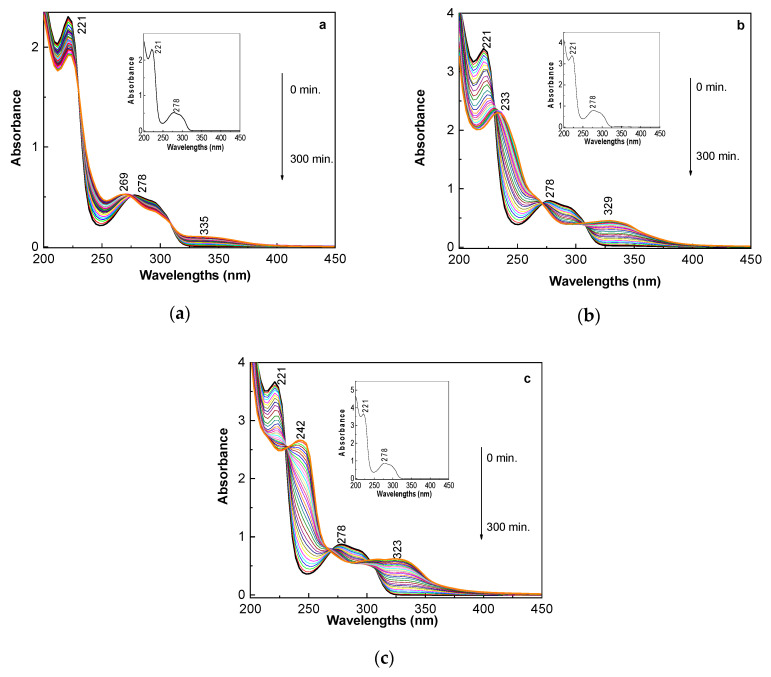
Ultraviolet-visible (UV-VIS) spectra of the samples labelled melatonin MEL-1 (**a**), MEL-2 (**b**), and MEL-3 (**c**) under exposure to ultraviolet (UV) light for up to 300 min. The UV-VIS spectra of the three samples before being exposed to UV light are shown in the inserts.

Figure 1 shows the UV-VIS spectra of the MEL-1, MEL-2, and MEL-3 samples before and after being exposed to UV light for 5 h. Before UV irradiation, the UV-VIS spectra of MEL alone and interacting with NaOH are characterized by two bands at 221 nm and 278 nm, respectively. In the case of the MEL-1 solution, the ratio between the absorbance of the bands at 221 nm and 278 nm is equal to 4.42. A change in the value of this ratio at 4.29 and 3.86 occurs when NaOH solutions, with concentrations of 1.5 and 3 mM, are added to the MEL-1 solution. Significant changes are noted when the three samples were exposed to UV light. In the case of the MEL-1 sample, as the exposure time to UV light increased to 5 h, one can observe a shift in the absorption band from 278 to 269 nm, which occurs simultaneously with the emergence of a new band in the 300–350 nm spectral range, whose absorbance gradually increases. After 5 h of exposure to UV light, the ratio between the absorbance of the bands localized in the 200–250 nm and 300–350 nm spectral ranges has a value of 19.3. The isosbestic points at 308, 275, and 230 nm indicate the production of new chemical compounds in the MEL-1 solution. According to Figure 1b,c, the MEL-2 and MEL-3 samples have a similar behavior as the exposure time to UV light increased to 5 h. This consists in the disappearance of the band at 278 nm, which occurs simultaneously with the appearance of the band localized in the 300–350 nm spectral range.

**Figure 2:**
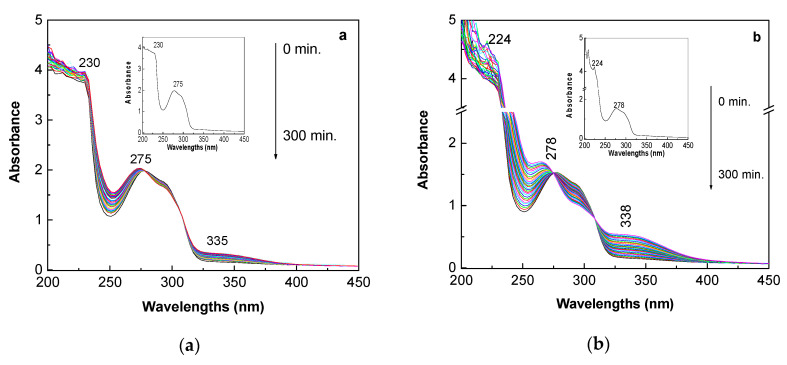
UV-VIS spectra of (**a**) MEL 0.7 mM obtained by the dispersion of a commercial tablet in water and (**b**) the sample obtained by the interaction of 2 mL of MEL 0.7 mM with 1 mL of NaOH 3 mM. Both samples were exposed to UV light for up to 300 min. The UV-VIS spectra of the two samples before being exposed to UV light are shown in the inserts.

In contrast to the MEL-1 spectral changes reported under exposure to UV light, a gradual up-shift of the band from 221 nm to 233 and 242 nm is highlighted in the case of the MEL-2 and MEL-3 samples, respectively, when the exposure time to UV light increases from 0 to 5 h. A careful analysis of the UV-VIS spectra of the MEL-2 and MEL-3 samples, recorded under exposure to UV light, indicates the appearance of two isosbestic points at (i) 308 nm and 272 nm and (ii) 302 nm and 269 nm, respectively. After 5 h of exposure to UV light, the ratio of the absorbance of the bands localized in the 200–250 nm and 300–350 nm spectral ranges becomes equal to 5.11 and 4.29 in the case of the MEL-2 and MEL-3 samples, respectively. Figure 2a,b highlight similar behavior of the commercial tablets of MEL in the absence and presence of NaOH during exposure to UV light for 5 h.

All these changes suggest the generation of new compounds in the MEL-2 and MEL-3 samples under UV light.

### 2.2. Photoluminescence as an Optical Tool for Highlighting MEL Photodegradation

**Figure 3:**
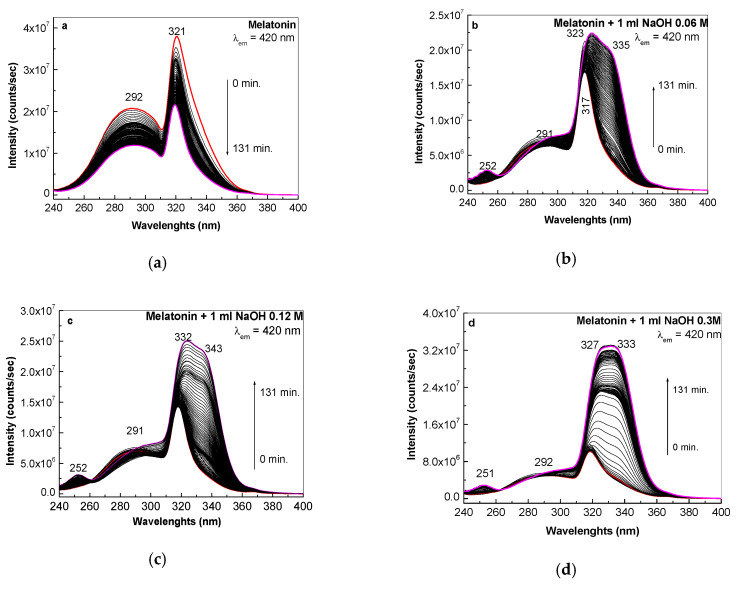

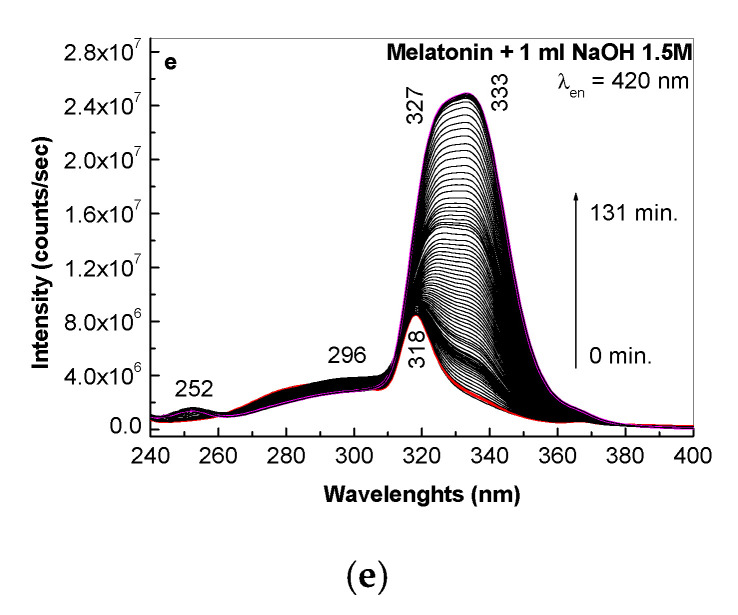
The photoluminescence excitation (PLE) spectra of the melatonin (MEL) solution (**a**) and the mixtures of the MEL solution with the NaOH solutions with concentrations equal to 0.06 (**b**), 0.12 (**c**), 0.3 (**d**), and 1.5 M (**e**) are recorded when the emission wavelength is 420 nm. The MEL solution was prepared using powder purchased from Aldrich-Sigma. The red curves indicate the PLE spectra of the samples before ultraviolet (UV) irradiation. The magenta curves show the PLE spectra of the samples after 131 min of exposure to UV light. The black curves correspond to the PLE spectra collected after 97 s of exposure to UV light. The total number of PLE spectra recorded in the case of each sample is 81.

Additional information concerning the MEL photodegradation in the absence and the presence of alkaline aqueous solutions is presented in the following PLE and PL studies. According to Figure 3a, the PLE spectrum of MEL is characterized by a high-intensity band with its maximum at 321 nm and a shoulder at around 292 nm. Under UV light, the intensity of the PLE band decreases from 3.7 × 10^7^ at 2.1 × 10^7^ counts/s. According to Figure 3b–e, in the absence of UV light, the intensity of the PLE spectra of the MEL samples that interacted with the NaOH solutions, with concentrations of 0.06, 0.12, 0.3, and 1.5 M, decrease at 1.6 × 10^7^, 1.4 × 10^7^, 1 × 10^7^, and 8 × 10^6^ counts/s, respectively. After exposure to UV light for 131 min, the PLE spectra intensity of the MEL samples, which have interacted with the NaOH solutions, with concentrations equal to 0.06, 0.12, 0.3, and 1.5 M, increase up to 2.2 × 10^7^, 2.4 × 10^7^, 3.25 × 10^7^, and 2.5 × 10^7^ counts/s, respectively. This change is accompanied by the broadening of the band localized in the 310–360 nm spectral range, which occurs simultaneously with the increase in the band intensity at 323 nm (Figure 3b–e). The origin of the PLE band at 323 nm (Figure 3) must be the same as that of the absorption band, which is reported to be localized in the 300–350 nm spectral range (Figure 1). According to Figure 3, the ratio of the intensities of the PLE bands localized in the 310–360 and 260–300 nm spectral ranges is equal to 1.82, 3.11, 3.35, 5.88, and 8.77, when the MEL solution has been exposed to UV light for 131 min with and without the interaction with the NaOH solutions at concentrations equal to 0.06, 0.12, 0.3, and 1.5 M, respectively. In addition to the changes reported in Figure 3, in the case of the MEL samples interacting with increasingly concentrated NaOH solutions, as the exposure time to UV light increases to 131 min, the emergence and gradual increase of the intensity of a new band at 252 nm is observed.

**Figure 4:**
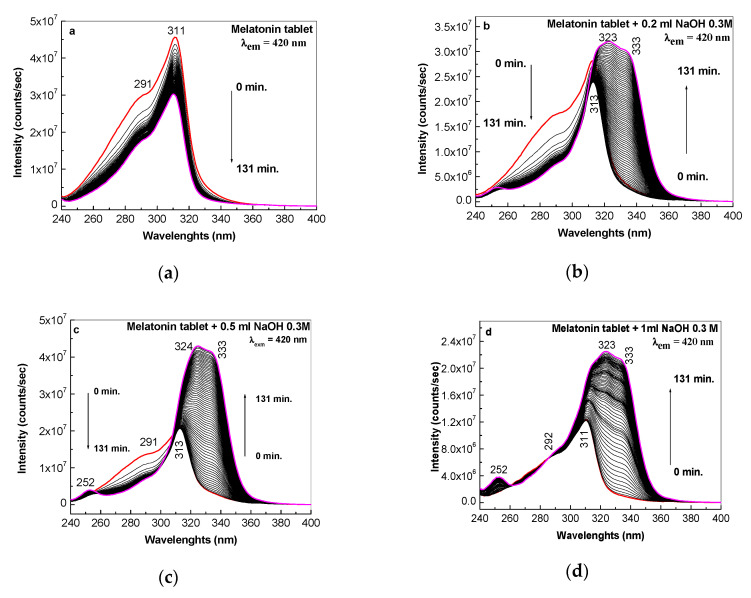

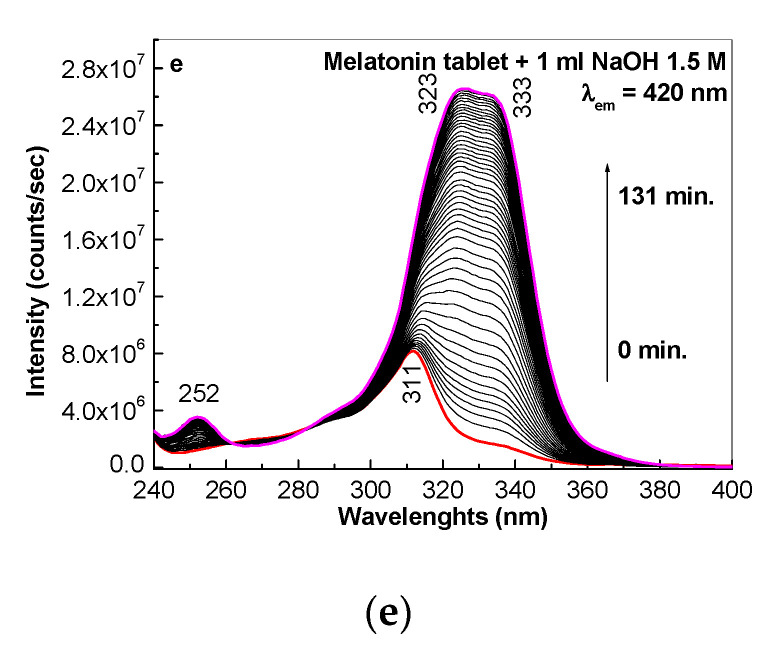
The PLE spectra of the MEL solution (**a**) and mixtures of MEL with 0.2 mL of NaOH 0.3 M (**b**), 0.5 mL of NaOH 0.3 M (**c**), 1 mL of NaOH 0.3 M (**d**), and 1 mL of NaOH 1.5 M (**e**) were recorded using the emission wavelength of 420 nm. The MEL solution was prepared using commercial tablets purchased from a local pharmacy. The red curves indicate the PLE spectra of the samples in the initial state. The magenta curves correspond to the PLE spectra of the samples after UV irradiation for 131 min. The black curves correspond to the PLE spectra collected after 97 s of exposure to UV light. The total number of PLE spectra recorded in the case of each sample is 81.

A similar behavior is demonstrated to take place in the case of the commercial MEL tablets, which have interacted with increasingly concentrated NaOH solutions, when the exposure time to UV light increases to 131 min (Figure 4).

**Figure 5:**
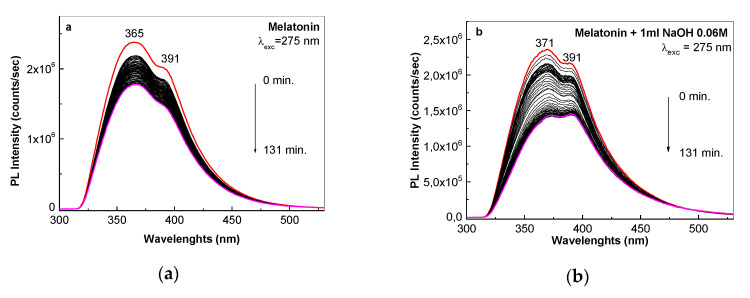

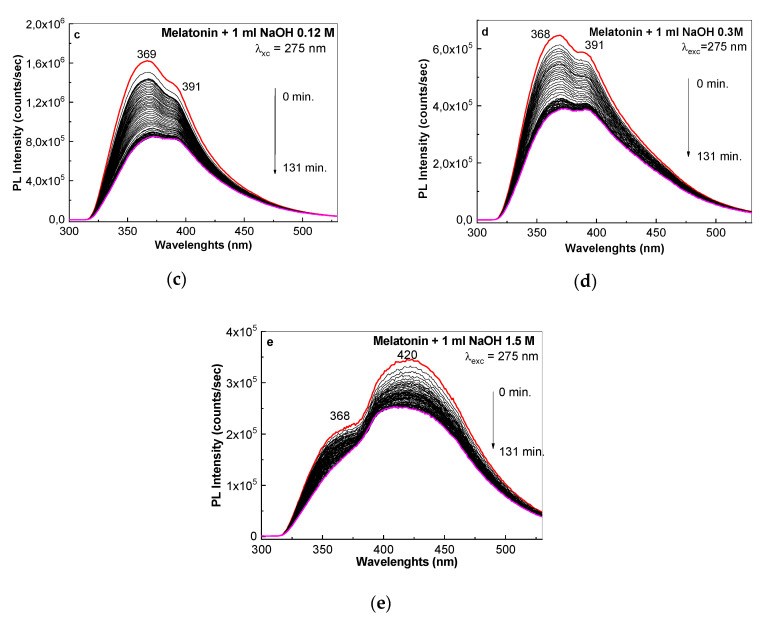
The photoluminescence (PL) spectra of the MEL solution (**a**) and mixtures of MEL with NaOH solutions with concentrations equal to 0.06 (**b**), 0.12 (**c**), 0.5 (**d**), and 1.5 M (**e**) were recorded at the excitation wavelength of 275 nm. The MEL solution was prepared using powder purchased from Aldrich-Sigma. The red curves indicate the PL spectra of the samples before exposure to ultraviolet (UV) light. The magenta curves correspond to the PL spectra of the samples after 131 min of UV irradiation. The black curves highlight the PL spectra collected after 130 s of exposure to UV light. The total number of PL spectra in the case of each sample is 60.

**Figure 6:**
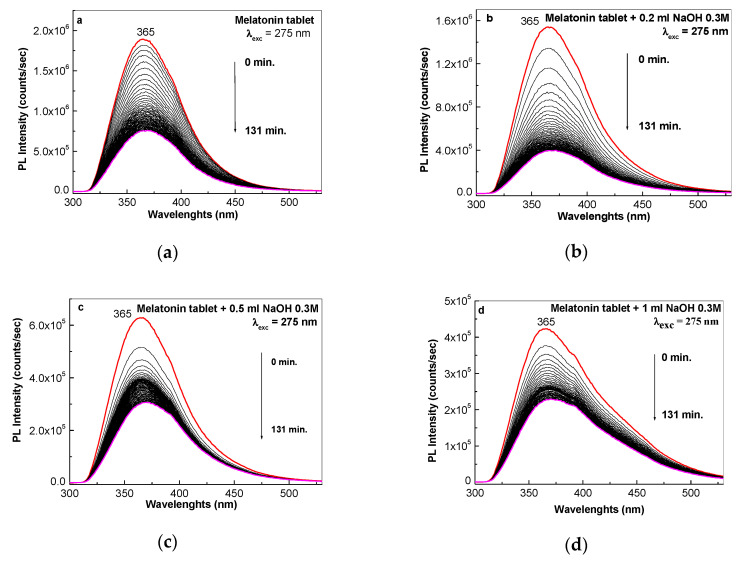

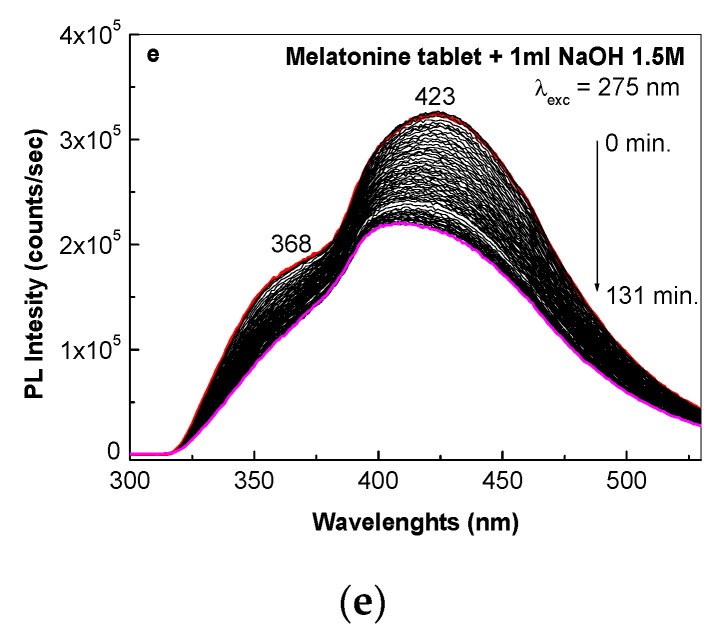
The PL spectra of the MEL solution (**a**) and mixtures of MEL with the NaOH solutions with concentrations equal to 0.06 (**b**), 0.12 (**c**), 0.5 (**d**), and 1.5 M (**e**) were recorded at the excitation wavelength of 275 nm. The MEL solution was prepared using commercial tablets purchased from a local pharmacy. The red curves indicate the PL spectra of the samples before exposure to UV light. The magenta curves highlight the PL spectra of the samples after 131 min of exposure to UV light. The black curves highlight the PL spectra recorded after 130 s of UV irradiation. The total number of PL spectra recorded in the case of each sample is 60.

Figure 5 and Figure 6 show the PL spectra of the MEL samples prepared using powder from the Aldrich-Sigma company and pharmaceutical tablets, respectively. According to Figure 5, the PL spectrum of MEL is characterized by an emission band at 365 nm, showing a shoulder at 391 nm, and the PL spectrum intensity is equal to 2.38 × 10^6^ counts/s. In the presence of aqueous solutions of NaOH 0.06, 0.12, 0.3, or 1.5 M, the MEL PL band intensity decreases to 2.36 × 10^6^, 1.62 × 10^6^, 6.47 × 10^5^, and 3.4 × 10^5^ counts/s, respectively. After the MEL sample was exposed to UV light for 131 min in the absence and presence of NaOH 0.06, 0.12, 0.3, or 1.5 M, a progressive decrease in the PL spectra intensity of MEL at 1.77 × 10^6^, 1.43 × 10^6^, 8.48 × 10^5^, 3.91 × 10^5^, and 2.49 × 10^5^ counts/s, respectively, occur. These variations are accompanied by an increase of the half-width of the emission band, as a result of the intensity increase of the new emission band at 420 nm.

The same changes can be easily noticed in the case of the samples prepared from commercial tablets, which contain both the active component, i.e., MEL, and excipients (Figure 6). This result indicates that excipients do not induce additional changes in comparison with those reported to have been induced by the active compound alone. In order to support these sentences and taking into account Figure 4 and Figure 6, Table 1 shows the intensities of the PLE and PL spectra of the commercial MEL tablets in the initial state and after the interaction with NaOH, depending on the exposure time to UV light.

**Scheme 1:**
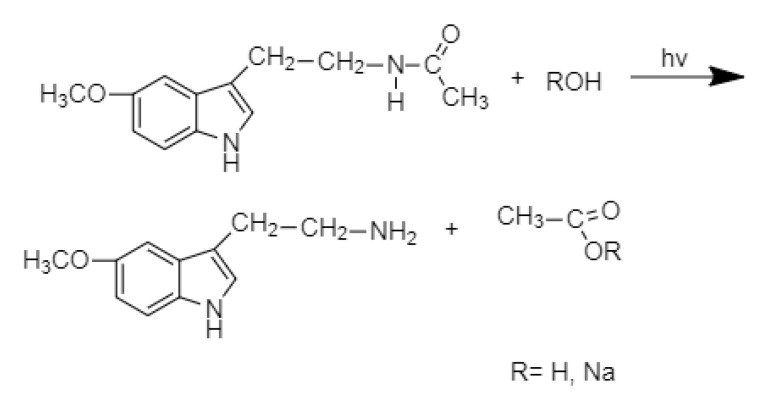
Photochemical reaction of melatonin (MEL) in the presence of NaOH.

In Figure 5 and Figure 6, the PL band with the maximum around 420–423 nm is noted only in the case of the NaOH solution with the highest concentration. In this stage of our investigation, to explain the presence of the emission band at 420–423 nm, is difficult. However, we think that this originates in the interaction of MEL with NaOH, when the formation of a larger amount of 5-methoxy tryptamine takes place. An explanation for these changes must take into account Scheme 1. According to Scheme 1, the reaction of MEL with NaOH leads to the formation of 5-methoxi tryptamine and sodium acetate.

### 2.3. Raman Scattering and IR Spectroscopy Studies Concerning the Photodegradation of MEL

Additional information on the reaction of MEL with NaOH is shown through the following correlated studies using Raman scattering and IR spectroscopy.

**Figure 7:**
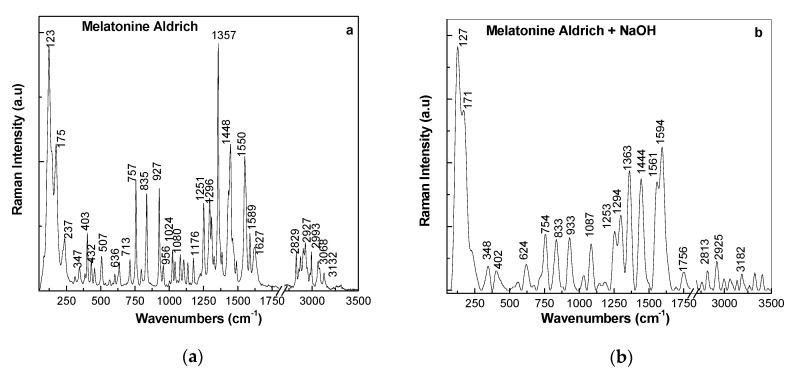
Raman spectra of MEL (**a**) before and (**b**) after interaction with NaOH.

**Figure 8:**
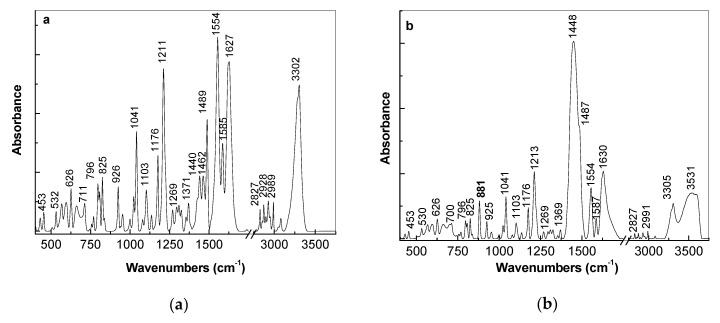
IR spectra of MEL (**a**) before and (**b**) after interaction with NaOH.

In order to support Scheme 1, in Figure 7, the Raman spectra of MEL, before and after its interaction with NaOH, are shown. According to Figure 7a, the Raman lines of MEL are situated at 347, 403, 507, 636, 713, 757, 835, 927, 956, 1024, 1080, 1176, 1251, 1296, 1357, 1448, 1550, 1589, 1627, and 2829–3132 cm^−1^, which are attributed to the vibrational modes of -COC twisting in anisole, CH_2_ twisting in indole acetamide, the in-plan bending of anisole, NH out-of-plane bending NH in acetamide amide IV, CH_2_ rocking in methylene, C-H out-of-plane bending in indole (pyrrole), C-H out-of-plan bending in indole (benzene), CCC bending mode in indole acetamide, phenyl breathing + CH_3_ rocking in anisole, ring-in-plane bending in indole, O-CH_3_ + pyrrole CCC bending in anisole-indole, CH_3_ out-of-plane rocking in acetamide anisole, CH_2_ wagging in methylene, NCH in-plane bending in indole, NH bending + CN stretching + C=C stretching in acetamide amide III, NH bending + C-N + C=O stretching in acetamide amide II, NH in-plane bending in the acetamide group, C-C ring stretching in indole, C=O stretching + C-N stretching + CCN deformation in acetamide amide I, and CH_3_ + CH_2_ + NH stretching [19]. The main changes observed in the Raman spectrum as a consequence of the interaction of MEL with NaOH are as follows: (i) a down-shift of the lines from 636, 1448, and 2829 cm^−1^ to 624, 1444, and 2813 cm^−1^, respectively, (ii) an up-shift of the lines from 927, 1080, 1357, 1550, and 1589 cm^−1^ to 933, 1087, 1363, 1561, and 1594 cm^−1^, respectively, and (iii) the presence of two new lines of a low intensity with the maximum at 1756 and 3002 cm^−1^. The Raman lines at 624, 1756, and 3002 cm^−1^ indicate the appearance of carboxylic salt of the type, CH_3_COONa [20,21]. Figure 8a highlights the main IR bands of MEL, which have their maxima at 453, 532, 626, 711, 796, 825, 926, 1041, 1103, 1176, 1211, 1269, 1371, 1440, 1489, 1554, 1585, 1627, 2827–2874–2928–2989, and 3302 cm^−1^. Figure 8 shows the significant changes that the interaction of MEL with NaOH induces. They are as follows: (i) the appearance of new IR bands at 881 and 3531 cm^−1^, (ii) a shift of the bands from 1440 to 1448 cm^−1^, which occurs simultaneously with the disappearance of the band at 1585 cm^−1^, (ii) the variation of the ratio between the absorbance of the bands localized in the 1400–1500, 1000–1250, and 1500–1650 cm^−1^ spectral ranges, i.e., A_1400–1500_/A_1000–1250_ and A_1400–1500_/A_1500–1650_, from 1.2 and 1.14 to 2.91 and 2.92, respectively, (iii) an increase in the width of the IR band in the field of small energies due to the NH_2_ scissoring vibrational mode (around 1650 cm^−1^), (iv) a significant decrease of the absorbance of the band at 3302 cm^−1^, which occurs simultaneously with the appearance of a new band with the maximum at 3531 cm^−1^. The IR bands at 881 and 3531 cm^−1^ are assigned to the vibrational modes of the out-of-plane NH bending and NH stretching in primary amine [22].

These changes confirm that the interaction of MEL with NaOH results in the generation of compounds of primary amine groups, as shown in Scheme 1.

### 2.4. X-ray Photoelectron Spectroscopy Studies on the Photodegradation of MEL

**Figure 9:**
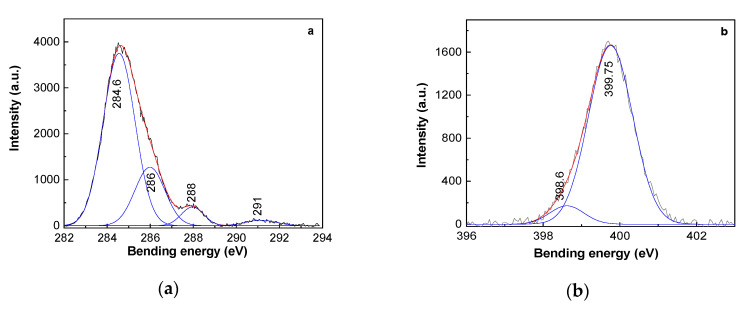
(**a**) The x-ray photoelectron spectroscopy (XPS) C1s and (**b**) N1s spectra of MEL.

**Figure 10:**
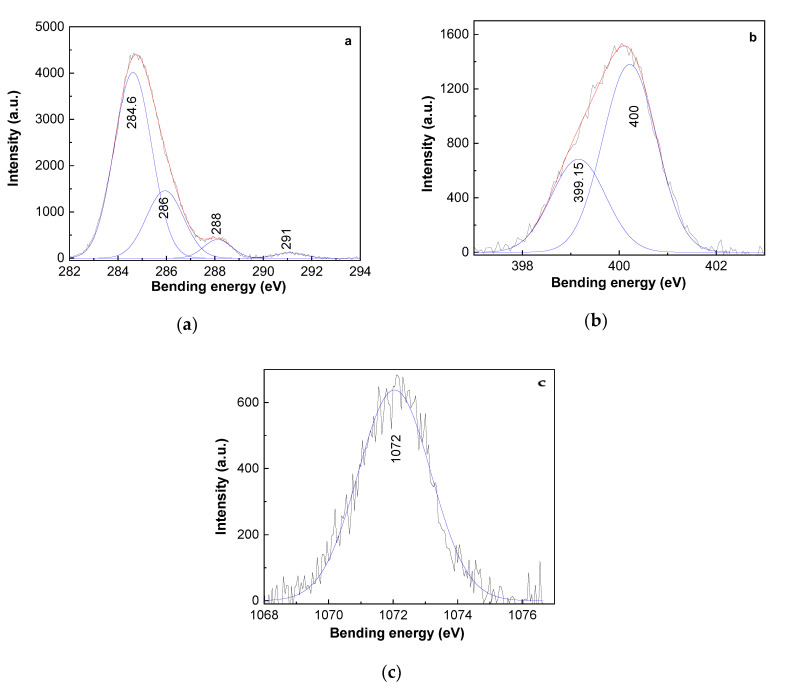
(**a**) The X-ray photoelectron spectroscopy (XPS) C1s, (**b**) N1s, and (**c**) Na1S spectra of melatonin (MEL) interacting with NaOH.

Figure 9 and Figure 10 show the XPS spectra of MEL, before and after the interaction with NaOH. Figure 9 first shows that, by the deconvolution of the XPS’ C1s spectrum of MEL, one observes an intense band at 284.6 eV, which shows a shoulder at 286 eV. The first peak is assigned to the sp^2^ C=C bond [23], while the peak at 286 eV can be attributed to the sp^3^ C-C, C-NH, and CO bonds, respectively [23]. The low-intensity peak at 288 eV was assigned to the C=O and -CO-NH bonds [24]. The very-low-intensity band at 291 eV was associated with the π−π* interactions in sp^2^ C=C bonds [25]. Second, Figure 9 shows an intense peak at 399.75 eV in the XPS N1s spectrum of MEL, which is accompanied by another low-intensity peak at 398.6 eV attributed to the amide group and the N atoms from the heterocycle of substituted tryptamine, respectively [26].

Any change is remarked in the XPS C1s spectrum of MEL interacting with NaOH. According to Figure 10, significant changes were induced in the case of the XPS N1s spectrum of MEL interacting with NaOH. In comparison with the XPS’ N1S spectrum of MEL (Figure 9), in Figure 10b, one observes (i) a shift of the two peaks at 399.15 and 400 eV, and (ii) a ratio between the intensities of the two peaks at 400 and 399.15 eV equal to 2. The peak at 400 eV is assigned to the NH bond in the alkylamine of the type, RNH_2_, where R corresponds to the alkyl group [27]. In our opinion, the peak at 400 eV confirms the first product of the reaction shown in Scheme 1.

Figure 10c shows a peak at 1072 eV in the XPS Na1s spectrum, which was reported to be characteristic of sodium acetate [28]. This is a compound that was reported to be produced in accordance with Scheme 1.

### 2.5. X-ray Diffraction Studies on the Photodegradation of MEL

**Figure 11:**
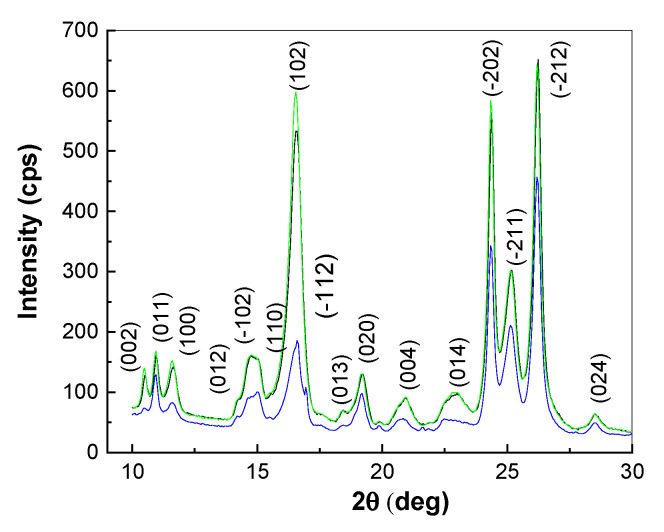
X-ray diffraction (XRD) patterns of MEL before (black curve) and after (green curve) 131 min of UV irradiation and MEL interacting with NaOH in the solid state, which was followed by exposure to UV light for 131 min (blue curve).

The structure of the MEL sample is characterized by X-ray diffraction measurements both in the initial state and after its reaction with NaOH. The diffraction patterns are shown in Figure 11.

In Figure 11, the XRD pattern of MEL shows a series of diffraction peaks, which can be easily identified according to the MEL’s powder diffraction file, PDF-00-055-1977. The relevant peaks are in the range of 10° < 2θ < 30° and are assigned to the crystalline planes (002), (011), (100), (012), (-102), (110), (102), (-112), (013), (020), (004), (120), (014), (-202), (-211), (-212), and (024). The interaction of MEL with UV light for 131 min induces only a slight increase in the intensity of the diffraction peak (102). In comparison with MEL in the initial state, significant changes in the XRD pattern of the MEL that interacted with NaOH in the solid state and was subsequently exposed to UV light for 131 min are highlighted by the increasing ratio between the intensities of the peaks attributed to the following crystalline planes: (i) (011), (002), and (100) and the I_(011)_/I_(002)_ and I_(011)_/I_(100)_ ratios varying from 1.36 and 1.17 to 2.26 and 1.85, respectively, and (ii) (-212), (-202), and (102) and the I_(-212)_/I_(202)_ and I_(-212)_/I_(102)_ ratios ranging from 1.16 and 1.24 to 1.36 and 2.73, respectively. A careful analysis of the XRD pattern of MEL in the range 15.5° < 2θ < 17.5° indicates the presence of a diffraction peak close to 17° in 2θ, which was not observed in the initial state of MEL. These changes indicate a preferential interaction of NaOH with MEL’s crystalline planes (002), (100), (102), and (-202).

## 3. Materials and Methods

Melatonin [N-acetyl-5-methoxytryptamine] and sodium hydroxide (NaOH) were purchased from Sigma Aldrich (St. Louis, MO, USA). The commercial MEL tablets, containing 3 mg of MEL, various excipients, like maize starch, pregelatinized starch, colloidal silicon dioxide, and lubricants, such as magnesium salts of fatty acids, were purchased from a local pharmacy. In this work, each MEL tablet was ground and dispersed in 3 mL of distilled water, ultra-sonicated for 10 min, and then filtered, which produced a clear solution.

A fresh solution of MEL in distilled water (1 mg/3 mL, noted as MEL-0) was prepared using powder purchased from Sigma Aldrich and subsequently diluted to a MEL concentration equal to 0.7 mM, which was labelled MEL-1.

In order to study MEL photodegradation by UV-VIS spectroscopy, two samples were prepared: a mixture of 2 mL of MEL 0.7 mM and 1 mL of NaOH aqueous solution, with concentrations equal to 1.5 and 3 mM, which were labelled MEL-2 and MEL-3, respectively. 

All three solutions were directly exposed to the UV light using a mercury lamp emitting at 257 nm.

The UV-VIS spectra of the samples labelled MEL-1, MEL-2, and MEL-3 were recorded with a Perkin Elmer UV-VIS-NIR spectrophotometer, Lambda 950 model, in the spectral range of 200–450 nm, with a scan rate of 96 nm/min. The UV-VIS spectra of each sample were recorded at intervals of 10 min of the UV irradiation with an overall time of 5 h.

The PL and photoluminescence excitation (PLE) spectra of an aqueous solution of MEL 1.29 mM and mixtures of the MEL with 1 mL of NaOH 0.06, 0.12, 0.3, or 1.5 M were recorded at room temperature with a Flurolog-3 spectrophotometer, FL3-221 model, from Horiba Jobin Yvon.

The Raman spectra of MEL in the initial state and after its reaction with NaOH were obtained using an FT Raman spectrophotometer, RFS100S model, from Bruker.

The IR spectra of MEL in the initial state and after its reaction with NaOH were obtained using an FTIR spectrophotometer, Vertex 80 model, from Bruker.

The XPS spectra of MEL in the initial state and after its reaction with NaOH were recorded using a SPECS spectrometer based on a Phoibos 150 electron energy analyzer, operating in a constant energy mode. An aluminum anode (Al Ka 1486.74 eV) was used as a monochromatic X-ray source. A wide range spectra over 1400 eV binding energies were recorded using 50 eV of pass energy. High-resolution spectra were acquired over 20 eV ranges at 10 eV of pass energy with an energy resolution of 0.7 eV. All the studies were performed in an ultra-high vacuum (10^−7^ Pa). The obtained data were analyzed using Spectral Data Processor SDPv7.0 software.

After being exposed to UV light for up to 310 min, a solid-state interaction of MEL (0.198 g) with NaOH (0.002 g) at a non-hydrostatic pressure of 0.58 GPa was performed. The structural properties of MEL, before and after its interaction with NaOH, were studied by XRD with a Bruker X-ray diffractometer, D8 Advanced model, using Cu_Kα_ radiation (λ = 1.54056 Å).

## 4. Conclusions

This work reports new results, obtained by UV-VIS spectroscopy and PL, concerning MEL hydrolysis in the presence of alkaline aqueous solutions and the photodegradation of MEL under UV light. Our results are as follows: (i) under UV light, the isosbestic point at 308 nm in the UV-VIS spectra of MEL in the absence and presence of NaOH indicates the emergence of new compounds, (ii) both in UV-VIS and PLE spectra, the new band localized in the 300–360 nm spectral range belongs to the compounds found to be the result of exposure to UV light, according to Scheme 1, and (iii) the interaction of MEL with NaOH and the photodegradation induced by UV light can be monitored by the variations in the positions and intensities of the PL and PLE bands. These changes are explained in consideration of the hydrolytic products of MEL. The main results concerning the hydrolytic products of MEL are shown by Raman scattering, XPS, and FTIR spectroscopy as follows: (i) the Raman lines at 624, 1756, and 3002 cm^−1^ belong to carboxylic salt of the type, CH_3_COONa, (ii) the peak at 1072 eV, in the XPS Na1s spectrum, is characteristic of CH_3_COONa, (iii) the IR bands at 881 and 3531 cm^−1^ can be attributed to the vibrational modes of out-of-plane NH bending and NH stretching in primary amine, which exist in 5-methoxytryptamine, and (iv) the peak at 400 eV in the XPS N1s spectrum assigned to the NH bond in alkylamine. We demonstrate that, in the case of the solid-state reaction of MEL with NaOH, the UV irradiation progressively induces a decrease in the intensity of the diffraction peaks at 10.4°, 11.6°, 16.5°, and 22.4°, which indicates a preferential interaction of NaOH with the MEL crystalline planes (002), (100), (102), and (-202).

## Figures and Tables

**Table 1:** The intensities of the PLE and PL spectra of the commercial MEL tablets in the initial state and after the interaction with NaOH, depending on the exposure time to UV light (C_MEL_, C_NaOH_, V_MEL_, V_NaOH_, t_UV_, I_PLE_, and I_PL_, corresponding to the MEL concentration, NaOH concentration, MEL volume, NaOH volume, time of the exposure to UV light, the intensity of the PLE spectrum, and the intensity of the PL spectrum).

C_MEL_ (mM)	V_MEL_ (mL)	C_NaOH_ (M)	V_NaOH_ (mL)	t_UV_ (min.)	I_PLE_ (counts/s)	I_PL_ (counts/s)
1.29	3	0	0	0	4.55 × 10^7^	1.89 × 10^6^
1.29	3	0	0	131	3.01 × 10^7^	7.49 × 10^5^
1.29	2.8	0.3	0.2	0	2.8 × 10^7^	1.54 × 10^6^
1.29	2.8	0.3	0.2	131	3.2 × 10^7^	3.95 × 10^5^
1.29	2.5	0.3	0.5	0	2.14 × 10^7^	6.28 × 10^5^
1.29	2.5	0.3	0.5	131	4.3 × 10^7^	3.06 × 10^5^
1.29	2	0.3	1	0	1.24 × 10^7^	4.23 × 10^5^
1.29	2	0.3	1	131	2.25 × 10^7^	2.29 × 10^5^
1.29	2	1.5	1	0	8.17 × 10^6^	3.25 × 10^5^
1.29	2	1.5	1	131	2.65 × 10^7^	2.19 × 10^5^
